# Tumor necrosis factor: is it time to change the name?

**DOI:** 10.1186/ar4541

**Published:** 2014-04-14

**Authors:** David S Pisetsky

**Affiliations:** 1 Medical Research Service, Durham Veterans Medical Center, 508 Fulton Street, Durham, NC 27705, USA; 2 Department of Medicine, Duke University Medical Center, Durham, NC 27705, USA

## 

Among targets of biological agents, tumor necrosis factor remains in the exclusive group that has retained its historical name - or at least one of them. Tumor necrosis factor is usually known by the three-letter abbreviation TNF and somehow has escaped a more bland and nondescript moniker such as IL-something. Endogenous pyrogen is now IL-1, B-cell stimulatory factor-2 is now IL-6, and T-cell growth factor is IL-2. But TNF is still TNF. The other name for TNF, cachectin, seems long gone, although arguably the metabolic actions of TNF are more relevant to the setting of inflammatory and autoimmune disease than a capacity to kill tumors. Although the name TNF has withstood the test of time, it depicts few of its myriad activities and little of its profound impact on the function of the brain, liver, and heart among other organs and tissues.

The designation TNF or tumor necrosis factor reflects the original discovery in the 1970s of a cytotoxic substance produced by immune cells stimulated by endotoxin. Since in animal models TNF kills tumors, investigators tried to exploit this action therapeutically in patients with cancer, but the administration of this cytokine made recipients too systemically sick. Though limiting TNF’s development in oncology, these studies revealed the powerful role of TNF in immunity. In addition to killing tumors, TNF can cause the metabolic derangements of cachexia as shown initially in studies on a novel factor produced by macrophages stimulated with bacterial or protozoal products. These studies called this factor cachectin. After studies showed that TNF and cachectin are the same molecule, only one name was possible and TNF became it.

With the recognition of TNF’s role in inflammation, studies focused on its potential as a target in the treatment of sepsis. Even though studies pointed to the efficacy of TNF blockade in animal models of sepsis, trials in humans were unsuccessful. Fortunately, these trials provided valuable reagents that led to the striking success of TNF blockade in the treatment of rheumatoid arthritis, ankylosing spondylitis, and Crohn’s diseases among many other inflammatory diseases. In the context of treating inflammation, the effects of TNF on cancer cells remain a concern as a side effect, with worries that a malignancy could emerge if TNF blockade impairs immune surveillance.

Trying to change TNF’s name at this point would be foolhardy and destined to fail if it eliminated the three-letter abbreviation. Knowing the power of name recognition, I would like to suggest a modest revision that would be more accurate while retaining the TNF nomenclature. Rather than having the N in TNF stand for necrosis, I think that it should stand for necroptosis. The brand identity thus remains intact.

Motivating consideration of this change is exciting research showing that the death form induced by TNF is better conceptualized as necroptosis than necrosis. For those readers who are not aficionados of death, necroptosis is a form of programmed cell death sometimes called programmed necrosis. Unlike classic necrosis (so-called accidental cell death), necroptosis is highly regulated and depends on the action of enzymes that internally deliver the death blow to a cell. As recent studies show, necroptosis is closely allied with apoptosis in the role of enzymes in cellular demise. Whereas the caspase enzymes mediate apoptosis, two enzymes called receptor-interacting protein (RIP) kinases 1 and 3 play a central role in killing cells by necroptosis by binding to form a necrosome.

The outcome of death via the caspases and RIP kinases is vastly different even though they can emerge from the same starting or check point and have cross-talk. (Be aware. Regulation here is very complex and pathways for activation and death are intertwined and criss-cross, resembling the diagram of a play in American football, with choices all along the way depending on the opposition.) In simple form, apoptosis renders a cell immunologically impotent. In contrast, necroptosis creates a cell that can induce inflammation. Indeed, recent studies suggest that the elaborate cleavage reactions and cellular rearrangements that characterize apoptosis have evolved not to kill cells but to strip away its immunological potential. In this sense, apoptosis may be a ‘good death’.

Reflecting the key role of cell injury and destruction in a host of diseases, the literature of cell death is now one of the liveliest in all of biology (pun intended) and is providing new insights into diverse processes that span the spectrum of medicine. Importantly, as these studies show, necroptosis, not necrosis, is truly the counterpart to apoptosis, and these two death forms are possible outcomes of stressors that include cytotoxic drugs, toxins, ischemia, or infection. With caspases inhibited, the cell can die by necroptosis and become more immunologically active. Interestingly, the venerable bioassay for TNF - the *in vitro* killing of the L929 fibrosarcoma line - is now one of the standard models for necroptosis, and caspase inhibition as well as treatment with TNF leads to this form of cell death.

A focus on necroptosis is not just a matter of semantics or intellectual disputation: it has important therapeutic implications. In oncology, an important goal in enhancing therapy is to boost killing by cytotoxic agents - whether chemotherapeutics, biologicals, or radiation - making them more lethal and rendering the dead cell more immunogenic. In contrast, in cardiovascular or cerebrovascular disease, the goal is the opposite: to prevent cell death from dangers such as ischemia and curtail the subsequent inflammation that can intensify injury and inflict bystander damage. Controlling, channeling, and commandeering death processes are therefore literally a matter of life and death in medicine.

Whereas necrosis, especially if caused by a harsh physical or chemical agent, is an explosion beyond the reach of therapy, necroptosis is a process and therefore can be contained. An agent named necrostatin 1 can block necroptosis by inhibiting the action of the RIP kinase 1 and its downstream effects. This agent and related analogues have shown impressive activity in models such as solid organ transplantation, ischemia-reperfusion injury, and acetaminophen toxicity. Acetaminophen toxicity has long been a model of necrosis. Guess what. The death form is more likely necroptosis than necrosis; as such, this important model of drug-induced liver injury can be inhibited by necrostatin 1 and other strategies to reduce RIP kinases.

The place of necroptosis in autoimmune and inflammatory disease is less certain since few studies have directly explored the role of this death form in immunopathogenesis. Right now, NETosis is hot but that form of death involves mostly neutrophils and other immune cells. Death is central to the pathogenesis of many rheumatological diseases, however. Muscle cells die in myositis, endothelial cells die in vasculitis, and glomerular cells die in nephritis. In general, these events have been called necrosis but, if they are more properly categorized as necroptosis, then the use of agents like necrostatin 1 could rescue and preserve tissue until glucocorticoids or immunosuppressives can more decisively halt the inflammation. In conditions such as rapidly progressing glomerulonephritis, an agent that stops injury could be the difference between renal failure and recovery. The time is now to make necroptosis a new watchword in rheumatology.

Had necroptosis been recognized when TNF was discovered, it is likely that this powerful cytokine would have been called tumor necroptosis factor. Although such a term is not as catchy as tumor necrosis factor (necroptosis’s four syllables are too bulky and jarring to come trippingly from the tongue), it provides a more clear and precise picture of the pathway induced and its consequences. By incorporating necroptosis into our thinking about inflammatory and autoimmune disease, new insights into the effects of TNF blockade should be possible and certainly could lead to the development of therapy directed at end-organ damage. In this case, death is not the end but the beginning, and its study gives new life to a critical domain of modern rheumatology.

## Competing interests

The author declares that he has no competing interests.

## Suggested Reading

Cho YS, McQuade T, Zhang H, Zhang J, Chan FK-M: **RIP1-dependent and independent effects of necrostatin-1 in necrosis and T cell activation.***PLoS One* 2011, **6:**e23209.

Duprez L, Takashi N, Van Hauwermeiren F, Vandendriessche B, Goossens V, Berghe TV, Declercq W, Libert C, Cauwels A, Vandenabeele P: **RIP kinase-dependent necrosis drives lethal systemic inflammatory response syndrome.***Immunity* 2011, **35:**908**–**918.

He S, Wang L, Miao L, Wang T, Du F, Zhao L, Wang X: **Receptor interacting protein kinase-3 determines cellular necrotic response to TNF-α.***Cell* 2009, **137:**1100**–**1111.

Kaczmarek A, Vandenabeele P, Krysko DV: **Necroptosis: the release of damage-associated molecular patterns and its physiological relevance.***Immunity* 2013, **38:**209**–**223.

Lau A, Wang S, Jiang J, Haig A, Pavlosky A, Linkermann A, Zhang Z-X, Jevnikar AM: **RIPK3-mediated necroptosis promotes donor kidney inflammatory injury and reduces allograft survival.***Am J Transplant* 2013, **13:**2805**–**2818.

Linkermann A, Hackl MJ, Kunzendorf U, Walczak H, Krautwald S, Jevnikar AM: **Necroptosis in immunity and ischemia-reperfusion injury.***Am J Transplant* 2013, **13:**2797**–**2804.

Linkermann A, Green DR: **Necroptosis.***N Engl J Med* 2014, **370:**455**–**465.

Ramachandran A, McGill MR, Xie Y, Ni H-M, Ding W-X, Jaeschke H: **Receptor interacting protein kinase 3 is a critical early mediator of acetaminophen-induced hepatocyte necrosis in mice.***Hepatology* 2013, **58:**2099**–**2108.

Takashi N, Duprez L, Grootjans S, Cauwels A, Nerinckx W, DuHadaway JB, Goossens V, Roelandt R, Van Hauwermeiren F, Libert C, Declercq W, Callewaert N, Prendergast GC, Degterev A, Yuan J, Vandenabeele P: **Necrostatin-1 analogues: critical issues on the specificity, activity and *in vivo *use in experimental disease models.***Cell Death Dis* 2012, **3:**e437.

Wu Y-T, Tan H-L, Huang Q, Sun X-J, Shen H-M: **zVAD-induced necroptosis in L929 cells depends on autocrine production of TNFα mediated by the PKC-MAPKS-AP-1 pathway.***Cell Death Differ* 2011, **18:**26**–**37.

